# Targeted mutation detection in breast cancer using MammaSeq™

**DOI:** 10.1186/s13058-019-1102-7

**Published:** 2019-02-08

**Authors:** Nicholas G. Smith, Rekha Gyanchandani, Osama S. Shah, Grzegorz T. Gurda, Peter C. Lucas, Ryan J. Hartmaier, Adam M. Brufsky, Shannon Puhalla, Amir Bahreini, Karthik Kota, Abigail I. Wald, Yuri E. Nikiforov, Marina N. Nikiforova, Steffi Oesterreich, Adrian V. Lee

**Affiliations:** 1 Department of Pharmacology and Chemical Biology, and Human Genetics, UPMC Hillman Cancer Center, Magee-Womens Research Institute, University of Pittsburgh, 204 Craft Avenue, Pittsburgh, PA 15213 USA; 2 Graduate Program in Integrated Systems Biology, University of Pittsburgh, Pittsburgh, USA; 3 Department of Pathology, Gundersen Health System, La Crosse, WI USA; 4 Department of Pathology, University of Pittsburgh, Pittsburgh, PA USA; 5 Department of Medicine, University of Pittsburgh, Pittsburgh, PA USA; 6 Department of Genetics and Molecular Biology, School of Medicine, University of Medical Sciences, Isfahan, Iran

**Keywords:** Breast cancer, Targeted sequencing, ctDNA, Clinical utility, Tumor burden

## Abstract

**Background:**

Breast cancer is the most common invasive cancer among women worldwide. Next-generation sequencing (NGS) has revolutionized the study of cancer across research labs around the globe; however, genomic testing in clinical settings remains limited. Advances in sequencing reliability, pipeline analysis, accumulation of relevant data, and the reduction of costs are rapidly increasing the feasibility of NGS-based clinical decision making.

**Methods:**

We report the development of MammaSeq, a breast cancer-specific NGS panel, targeting 79 genes and 1369 mutations, optimized for use in primary and metastatic breast cancer. To validate the panel, 46 solid tumors and 14 plasma circulating tumor DNA (ctDNA) samples were sequenced to a mean depth of 2311× and 1820×, respectively. Variants were called using Ion Torrent Suite 4.0 and annotated with cravat CHASM. CNVKit was used to call copy number variants in the solid tumor cohort. The oncoKB Precision Oncology Database was used to identify clinically actionable variants. Droplet digital PCR was used to validate select ctDNA mutations.

**Results:**

In cohorts of 46 solid tumors and 14 ctDNA samples from patients with advanced breast cancer, we identified 592 and 43 protein-coding mutations. Mutations per sample in the solid tumor cohort ranged from 1 to 128 (median 3), and the ctDNA cohort ranged from 0 to 26 (median 2.5). Copy number analysis in the solid tumor cohort identified 46 amplifications and 35 deletions. We identified 26 clinically actionable variants (levels 1–3) annotated by OncoKB, distributed across 20 out of 46 cases (40%), in the solid tumor cohort. Allele frequencies of ESR1 and FOXA1 mutations correlated with CA.27.29 levels in patient-matched blood draws.

**Conclusions:**

In solid tumor biopsies and ctDNA, MammaSeq detects clinically actionable mutations (OncoKB levels 1–3) in 22/46 (48%) solid tumors and in 4/14 (29%) of ctDNA samples. MammaSeq is a targeted panel suitable for clinically actionable mutation detection in breast cancer.

**Electronic supplementary material:**

The online version of this article (10.1186/s13058-019-1102-7) contains supplementary material, which is available to authorized users.

## Background

Advanced breast cancer is currently incurable. Selection of systematic therapies is primarily based on clinical and histological features and molecular subtype, as defined by clinical assays [1]. Large-scale genomic studies have shed light into the heterogeneity of breast cancer and its evolution to advanced disease [2, 3] and, coupled with the rapid advancement of targeted therapies, highlight the need for more sophisticated diagnostics in cancer management [4].

Next-generation sequencing (NGS)-based diagnostics allow clinicians to identify specific putative driver events in individual tumors. Correctly identifying disease drivers may enable clinicians to better predict treatment responses, and significantly improve patient care [5]. However, to date, the use of NGS in clinical diagnostics remains limited [6]. Published data regarding prognostic utility, and utilization for selection of targeted therapies or enrollment in clinical trials, is far from comprehensive.

The original 46 gene AmpliSeq Cancer Hotspot Panel (ThermoFisher Scientific) was shown to have a diagnostic suitability in primary lung, colon, and pancreatic cancers [7]; however, our previous report that surveyed the clinical usefulness of the 50 gene AmpliSeq Cancer Hotspot Panel V2 in breast cancer found that the panel lacks numerous known key drivers of advanced breast cancer [8]. For example, the panel does not include any amplicons in *ESR1*, which harbor mutations which are known to contribute to hormone therapy resistance (for review see [9]), and lacks coverage of the majority of known driver mutations in *ERBB2* [10].

The lack of any reported breast cancer-specific diagnostic NGS test inspired the development of MammaSeq™, an amplicon-based NGS panel built specifically for use in advanced breast cancer. We hypothesized that a breast cancer-specific test may offer a method for identifying therapeutic targets in solid tumor and circulating tumor DNA (ctDNA). Forty-six solid tumor samples from women with advanced breast cancer, plus a separate cohort of 14 samples of circulating tumor DNA (ctDNA) from 7 patients with metastatic breast cancer, were used in this pilot study to define the clinical utility of the panel. The patient cohort encompassed all 3 major molecular subtypes of breast cancer (luminal, ERBB2 positive, and triple negative) and both lobular and ductal carcinomas (Table 1).

**Table 1:** Patient and specimen characteristics

	Patients with available tumor tissue (*n* = 46)	Patients with available blood samples (*n* = 7)
Age		
Median age (years)	45	53
Range (years)	31–71	24–62
Race		
White	45 (97.8%)	7 (100.0%)
Black	1 (2.2%)	0 (0.0%)
Site		
Primary	10 (21.7%)	0 (0.0%)
Metastatic	36 (78.3%)	7 (100.0%)
Stage (Dx)		
I	10 (21.7%)	0 (0.0%)
II	8 (17.4%)	3 (21.4%)
III	13 (28.3%)	0 (0.0%)
IV	4 (8.7%)	2 (14.3%)
Unknown	11 (23.9%)	2 (14.3)
Hormone-receptor		
HR+ and HER2–	19 (41.3%)	2 (28.6%)
HR+ and HER2+	5 (10.9%)	0 (0.0%)
HR+ and HER2 unknown	1 (2.2%)	0 (0.0%)
HR– and HER2+	1 (2.2%)	0 (0.0%)
HR– and HER2–	17 (36.9%)	0 (0.0%)
Both unknown	2 (4.3%)	5 (71.4%)
Histopathology		
Ductal	34 (73.9%)	7 (100%)
Lobular	5 (10.9%)	0 (0.0%)
Mixed	3 (6.5%%)	0 (0.0%)
Other/unknown	4 (8.7%)	0 (0.0%)

## Methods

This report adheres by the REporting recommendations for tumour MARKer prognostic studies (REMARK) [11] where applicable. The methods for ctDNA isolation, processing, and analysis are in concordance with state-of-the-art approaches cited by the recent joint review from the American Society of Clinical Oncology and the College of American Pathologists [﻿12﻿].

### Patient sample collection

For MammaSeq NGS testing, this study utilized breast tumors from 46 patients and a separate cohort of blood samples from 7 patients. The research was performed under the University of Pittsburgh IRB approved protocol PRO16030066. The general patient characteristics are shown in Table 1, and more detailed patient information is shown in Additional file 1: Table S1. We utilized 46 of the 48 breast cancer cases previously described in a report by Gurda et al. [﻿8﻿]. These cases previously underwent AmpliSeq Cancer Hotspot Panel V2 (ThermoFisher Scientific) NGS testing between January 1, 2013, and March 31, 2015, within the UPMC health system. MammaSeq™ was performed on the DNA originally isolated from these tumor specimens and that was originally used for initial clinical testing [﻿8﻿]. Two cases were excluded due to insufficient DNA. In addition, a separate cohort of 7 patients with metastatic breast cancer (MBC) had 20 ml venous blood drawn in Streck Cell-Free DNA tubes between July 1, 2014, and March 29, 2016. All patients signed informed consent, and samples were acquired under the University of Pittsburgh IRB approved protocol (IRB0502025). We previously reported on the detection of ESR1 mutations in ctDNA from these 7 patients using droplet digital PCR (ddPCR) [13]. Serial blood draws (range 2–5) were available for 4 patients. A total of 14 blood samples from 7 patients were utilized for ctDNA, buffy coat DNA isolation, and NGS testing followed by ddPCR.

### Patient sample processing

Blood was processed as described previously [13]. Briefly, venous blood was drawn into leukocyte-stabilizing Streck tubes and processed to separate plasma and buffy coat by double centrifugation within 4 days of blood collection. One milliliter to 4 ml of plasma was used for isolation of ctDNA using QIAamp Circulating Nucleic Acid kit (Qiagen). ctDNA was quantified using Qubit dsDNA HS assay kit (ThermoFisher Scientific). Germline DNA (gDNA) was isolated from buffy coat using DNeasy Blood & Tissue Kit (Qiagen) for use as germline DNA control. gDNA was quantified using Qubit dsDNA BR assay kit (ThermoFisher Scientific). Patient-matched ctDNA and gDNA from the same tube were sequenced to allow subtraction of germline variants and identify somatic variants in ctDNA.

### Ion torrent sequencing

Twenty nanograms of DNA (10 ng per amplicon pool) was used for library preparation using Ion AmpliSeq™ Library Kit 2.0 (ThermoFisher Scientific) and the custom designed MammaSeq™ primer panel (Additional file 2: Data file 1). Template preparation by emulsion PCR and enrichment was performed on the Ion OneTouch 2 system (ThermoFisher Scientific). Template-positive Ion Sphere particles (ISP) were loaded onto Ion chips and sequenced. Patient-matched ctDNA and gDNA from the same tube were sequenced. ctDNA was sequenced using P1 chips (60 million reads) on the Ion Proton™ (ThermoFisher Scientific) at empirical depths of 1000× and 5000×, respectively. gDNA DNA was sequenced using 318 chip (6 million reads) on the Ion Torrent Personal Genome Machine (PGM™, ThermoFisher Scientific) at 500×.

### Variant calling

**Fig. 1:**
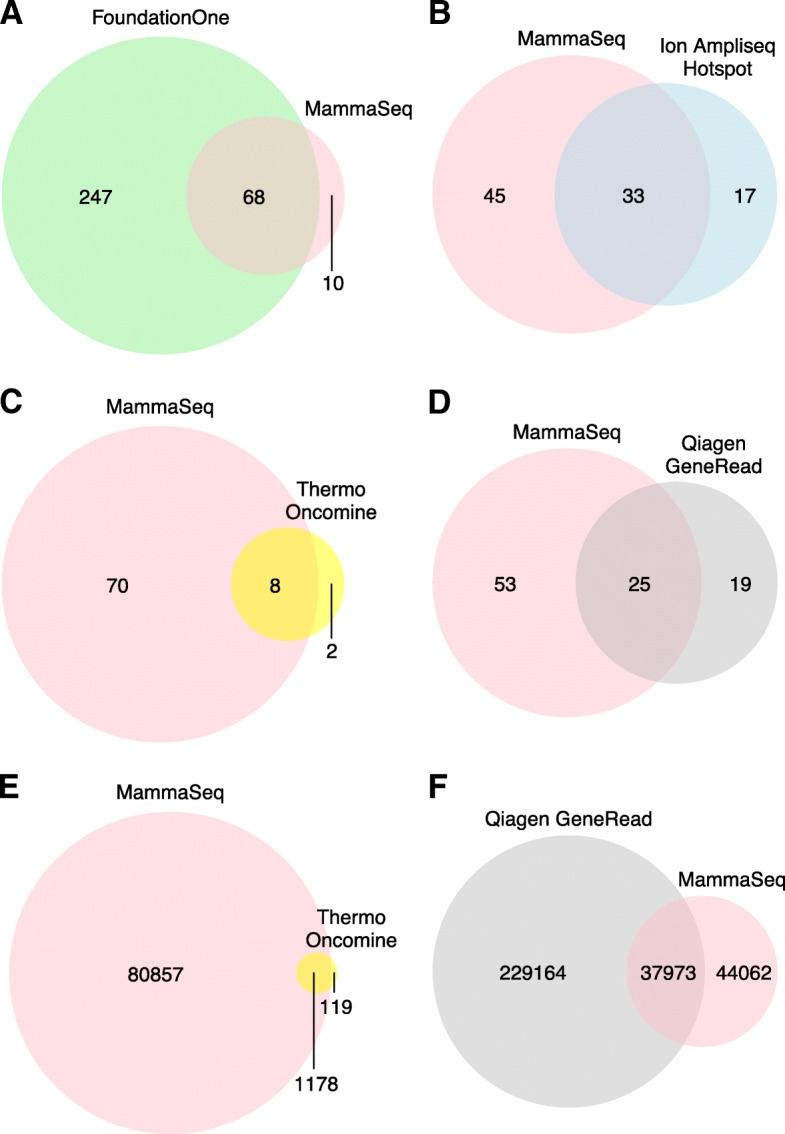
Coverage overlap between MammaSeq™ and select commercially available panels used in breast cancer. Overlap of genes present in the MammaSeq™ panel and the **a** Foundation Medicine FoundationOne panel, **b** Thermo Ion AmpliSeq Cancer Hotspot Panel (v2), **c** Thermo Oncomine Breast ctDNA Assay, and **d** Qiagen GeneRead Human Breast Cancer Panel. Overlap of the number of base pairs covered for the **e** Thermo Oncomine Breast ctDNA Assay and the **f** Qiagen GeneRead Human Breast Cancer Panel was calculated as the exact panel designs are publicly available

Ion Torrent Suite V4.0 was used to align raw fastq files to the hg19 reference genome and generate VCF files (4.0% AF cutoff for tumor samples, 1.0% AF cutoff for ctDNA samples). Raw sequence files are available upon request for those wishing to map data to Ch38. Cravat CHASM-v4.3 (http://hg19.cravat.us/CRAVAT/) was used to annotate variants with resulting protein changes and SNP annotation from ExAC [14] and 1000Genomes [15]. Variant calls from patient-matched gDNA (gDNA isolated from the same blood sample as the ctDNA) were used to remove germline variants from the 14 ctDNA samples in a patient-matched manner. SNP and sequencing artifact filtering, data organization, and figure preparation were performed in R (v3.4.2). The R package ComplexHeatmaps was used to generate Figs. 1 and 3a. CNVKit was used to call copy number across all genes; however, only genes containing more than 3 amplicons were reported (Table 2) [16]. DNA from the buffy coat of the ctDNA cohort was used to generate a single copy number reference which was used as a baseline for copy number calling on the solid tumor cohort. CNKit reports copy number as a log2 ratio change. CNV were reported if the absolute copy number was above 6 (log2(6/2) = 1.58) or below 1 (log2(1/2) = − 1).

**Table 2:** Seventy nine genes incorporated in the MammaSeq^TM^ gene panel

ABL1	CDK6	FGFR3	KDR	NOTCH1
AKT1	CDKN1B	FGFR4	KIT	NRAS
AKT3	CDKN2A	FOXA1	KMT2C	PAK1
ALK	CDKN2B	GATA3	KRAS*	PDGFRA
AR	CTCF	GRB7	MAP2K4	PIK3CA
ARID1A	CTNNB1	HIST2H2BE*	MAP3K1	PIK3R1
ATM	DNAH14	HRAS*	MAP3K4	PTCH1
AURKA	EGFR	IDH1*	MDM2	PTEN
AURKB	ERBB2	IGF1R	MDM4	RB1
BRAF	ERBB3	IKBKB	MET	RET
BRCA1	ERBB4	IKBKE	MTOR	RPTOR
BRCA2	ESR1	INPP4B	MYC	RUNX1
CCND1	EZH2*	INSR	NCOA3	SMO
CCNE1	FGF19	JAK2	NCOR1	STK11
CDH1	FGFR1	JAK3	NCOR2	TP53
CDK4	FGFR2	JUN*	NF1	

*Genes with less than 3 amplicons, for which copy number changes were not reported

### Data and code

Annotated, unfiltered, mutation, and CNV data, along with R code related to this study, are deposited on GitHub (https://github.com/smithng1215).

### ddPCR

Two nanograms of ctDNA or buffy coat DNA was subjected to targeted high-fidelity preamplification for 15 cycles using custom-designed primers (Additional file 3: Table S2) and PCR conditions previously described [13]. Targeted preamplification products were purified using QIAquick PCR Purification kit (Qiagen) and diluted at 1:20 before use in ddPCR reaction. 1.5 μl of diluted preamplified DNA was used as input for ddPCR reaction. ddPCR was performed for ESR1-D538G, FOXA1-Y175C, and PIK3CA-H1047R mutations. Custom ddPCR assays were developed for ESR1-D538G (Integrated DNA Technologies) and FOXA1-Y175C (ThermoFisher Scientific). Sequences are described in Additional file 4: Table S3. PIK3CA-H1047R was analyzed using PrimePCR ddPCR assay (Bio-Rad Laboratories) dHsaCP2000078 (PIK3CA)/dHsaCP2000077 (H1047R). Nuclease-free water and buffy coat-derived wildtype germline DNA as negative controls, and oligonucleotides carrying mutation of interest or DNA from a cell line with mutation as positive controls, were included in each run to eliminate potential false-positive mutant signals. An allele frequency of 0.1% was used as a lower limit of detection.

### Statistical analysis

All statistical analysis was performed in R 3.4.2. To determine if there was a significant correlation between mutational burden and copy number burden, we calculated the Pearson correlation coefficient between the number of somatic mutations in each sample, with the number of significant copy number changes in each sample. We did not examine a relationship between mutations and patient outcome due to the small sample size.

## Results

### Development of MammaSeq™ panel

To build a comprehensive list of somatic mutations in breast cancer, we combined mutation calls from primary tumors in TCGA (curated list level 2.1.0.0), and limited studies focused on metastatic breast cancer [17–19]. The biological function and druggability of mutated genes were investigated via Gene Ontology (GO) [20] and DGIdb (v2.0) databases [21]. The information regarding FDA approved drugs was downloaded from “https://www.fda.gov/Drugs” and added to our list. We used the following criteria to prioritize the clinically important mutated genes:

- The mutated gene is among significantly mutated genes (SMGs) in primary and metastatic samples.
- The mutated gene is clinically actionable (e.g., there is available FDA-approved drug(s) against it).
- The mutated gene is of functional importance in cancer (e.g., kinase genes were scored higher in the list).
- The mutation has been found in more than 5 primary tumors OR 2 metastatic tumors.
- The mutation has been found in both primary and metastatic lesions.

The final mutation list was then curated and narrowed down to 80 genes and 1398 mutations. Additional amplicons were added to select genes to ensure sufficient coverage of genes known to harbor functional copy number variants. Amplicon probe design was unsuccessful for 29 mutations, including all 3 mutations in the gene HLA-A, yielding a final panel consisting of 688 amplicons targeting 1369 mutations across 79 genes (Table 2). The percentage of each gene covered is shown in Additional file 5: Figure S1. Panel design is described in Additional file 2: Data file 1).

The panel includes 34 of the 50 (68%) genes incorporated in AmpliSeq Cancer Hotspot Panel V2 (Fig. 1). Genes that were not mutated in breast cancer (TCGA and in-house data) and genes that were not considered to be clinically actionable were not included. The MammaSeq™ panel includes 8 of the 10 (80%) genes and ~ 91% of the hotspots targeted by the Thermo Oncomine Breast ctDNA assay. MammaSeq™ covers 14% of the base pairs covered by the Qiagen Human Breast Cancer GeneRead DNAseq Targeted Array; however, it covers hotspots in over half of the genes (57%, plus an additional 34 genes). Of these panels, MammaSeq is the only one that includes CDK4 and CDK6, both of which can be targeted with FDA-approved CDK4/6 inhibitors [22]. Additional genes unique to MammaSeq include common drivers, CCND1, MTOR, and FGFR4. Finally, MammaSeq covers 68 of 315 genes targeted by the larger pan cancer Foundation Medicine, FoundationOne panel. Figure 1 details the overlap in coverage between MammaSeq™ and abovementioned commercially available panels.

### Characterization of genetic variants detected by MammaSeq in a solid tumor cohort

**Fig. 2:**
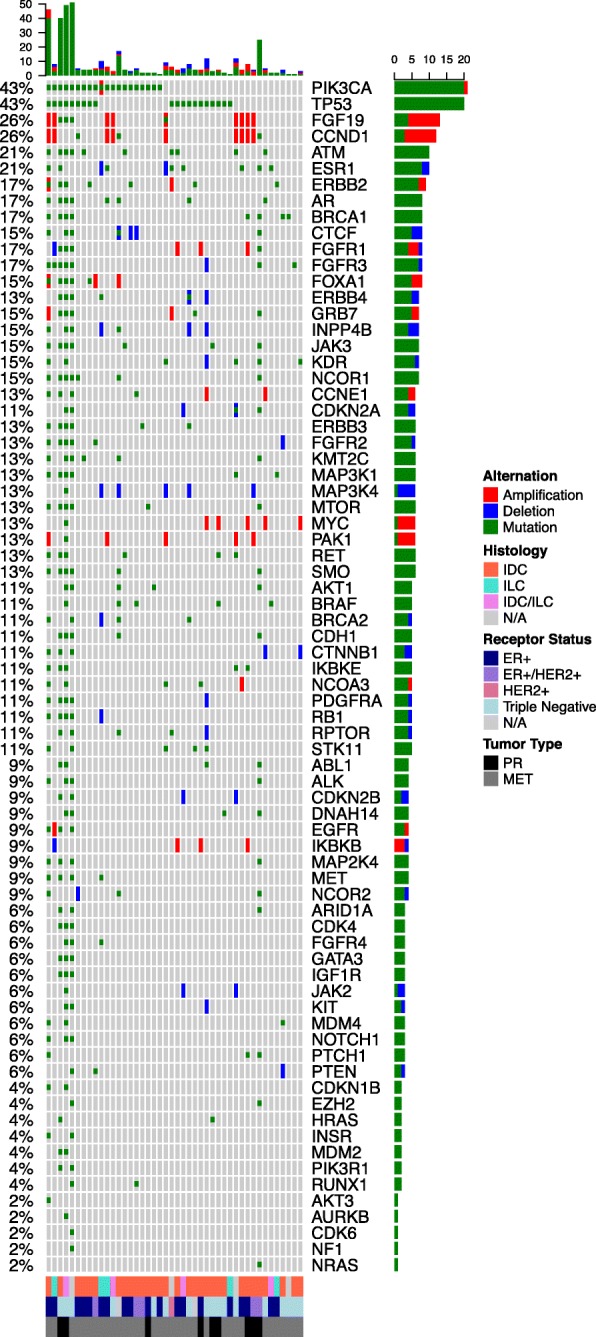
Genetic alterations identified by the MammaSeq™ gene panel in a test cohort of 46 breast cancers. Oncoprint depicting the distribution of somatic mutations, copy number amplifications (absolute copy number greater than 6), and deletions (absolute copy number less than 1)

To evaluate performance in mutation detection by the MammaSeq™ panel, sequencing was carried out on a cohort of 46 solid tumor samples, with a mean read depth of 2311× (Additional file 6: Figure S2). Four thousand nine hundred seventy total variants (mean 106, median 82) were called across all patient samples. We removed identical germline  variants that were present in more than 10 samples as these were likely to be platform-specific sequencing artifacts or common SNPs. Removing non-coding and synonymous variants yielded 1433 and 901 variants, respectively. To filter out less common polymorphisms, we removed variants annotated in ExAC [14] or the 1000Genomes [15] databases in more than 1% of the population. We removed variants with an allele frequency above 90%, as these were likely germline. Finally, to focus on high confidence mutations, we removed variants with a strand bias outside of the range of 0.5–0.6, yielding a total of 592 protein coding mutations (mean 12.9, median 3, IQR 3) (Fig. 2, Additional file 7:Data file 2). Of the variants (*n* = 119) previously reported by Gurda et al. on the same cases [8], > 98% were detected by MammaSeq. Analyzing the variant allele frequencies detected by both assay, we found an outstanding correlation (*R*^2^ = 0.98) (Additional file 8: Figure S3).

Interestingly, as noted by the variation between the mean and median, the total number of mutations was skewed toward a subset of samples (Fig. 2 top panel). Four hundred eight of the 592 mutations (69%) were found in just 4 of the 46 samples (Additional file 9: Figure S4). These 4 samples are outliers, as they are all more than 1.5 times the IQR plus the median. Counting of tumor infiltrating lymphocytes has not been performed on this cohort and is warranted to support the hypothesis that these tumors have high immune infiltrate and thus may respond to immune therapy. Three of these 4 samples with high mutational burden were of triple negative subtype, while the fourth was ER+/HER2+. The most common mutated genes were TP53 (57%) and PIK3CA (43%). We also noted common mutations in ESR1 (21%), ATM (21%), and ERBB2 (17%).

To examine CNV changes, we established a baseline for pull down and amplification efficiency by performing MammaSeq™ on normal germline DNA from 14 samples (7 patients—6 additional). CNVkit [16] was used to pool the normal samples into single reference and then call CNV in the solid tumor cohort (Fig. 1). CNV were identified in many common oncogenes including *CCND1*, *MYC*, and *FGFR1*. Two of the 3 *ERBB2*+ samples (via clinical assay) showed CNV by MammaSeq. FGF19 and CCND1 were co-amplified in 9 of the 46 (20%) solid tumors. Both genes are located on 11q13, a band identified in GWAS as harboring variants, including amplifications, associated with ER+ breast cancers [23]. There was not a correlation between mutational burden and copy number burden (Pearson correlation *p* value = 0.7445).

### Clinical utility of genetic variants detected by MammaSeq

**Fig. 3:**
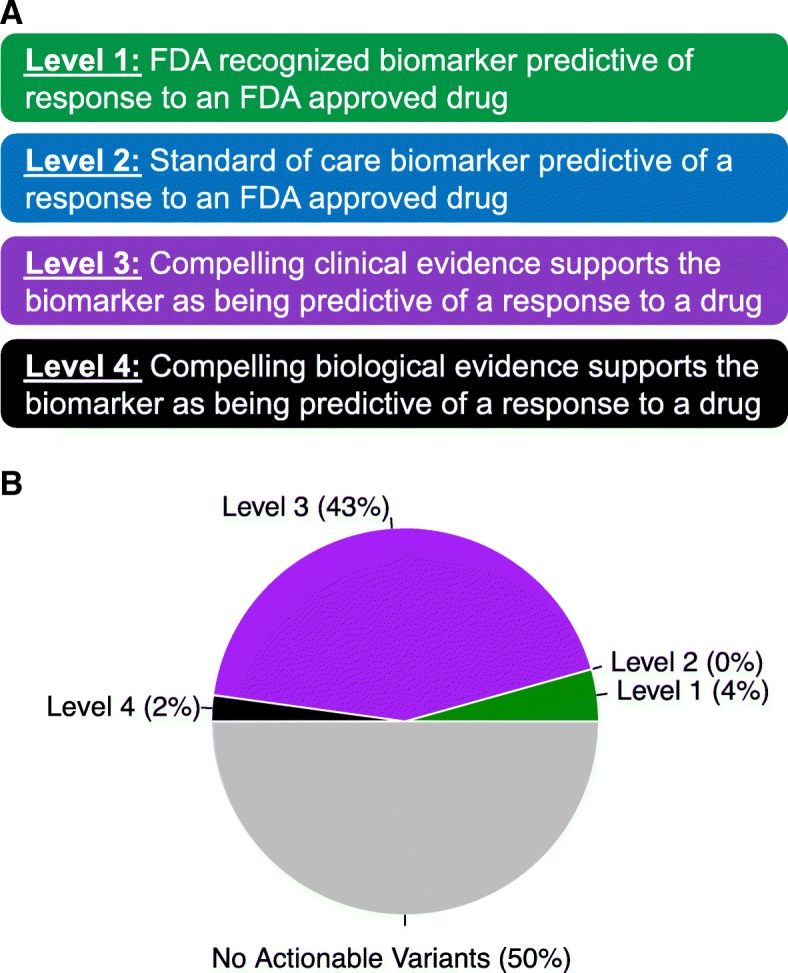
Clinical actionality of MammaSeq™ identified somatic alterations. **a** Annotation levels, adapted from OncoKB [25]. **b** Samples were categorized based on the most actionable alteration. Specific alterations and associated drugs are depicted in Table 3

To determine how many of the mutations have putative clinical utility, we utilized the OncoKB precision oncology knowledge database [24]. Twenty-five of the genes in the MammaSeq™ panel (32% of the panel) harbor clinically actionable variants with supporting clinical evidence (OncoKB levels 1–3). In total, we identified 28 actionable variants (26 single nucleotide variants (SNVs) and 2 ERBB2 amplifications) that have supporting clinical evidence (levels 1–3) and an additional 3 actionable variants supported by substantial research evidence (level 4) in the solid tumor cohort (Table 3). The 26 SNVs were distributed across 20 of the 46 cases (43%) (Fig. 3). Consistent with the report detailing the development of the OncoKB database [25], the vast majority of actionable variants in breast cancer are annotated at level 3, indicating that variants have been used as biomarkers in clinical trials; however, they are not FDA approved. In fact, the only level 1 annotated variant in breast cancer is *ERBB2* amplification.

**Table 3:** Identified variants in annotated in OncoKB with corresponding targeted therapeutics

Sample ID	Gene	Protein sequence change	Allele frequency	Level	Drugs
MET_03	ERBB2	Amplification	–	1	Lapatinib + trastuzumab, pertuzumab + trastuzumab, ado-trastuzumab emtansine, lapatinib, trastuzumab
MET_33	ERBB2	Amplification	–	1	
MET_39	AKT1	E17K	0.25	3	AZD5363
MET_18	ERBB2	I654V	0.122222	3	Neratinib
MET_32	ERBB2	I654V	0.461731	3	
MET_49	ERBB2	I654V	0.495495	3	
MET_07	ESR1	D538G	0.477717	3	AZD9496, fulvestrant
MET_21	ESR1	D538G	0.335884	3	
MET_28	ESR1	D538G	0.454271	3	
MET_27	ESR1	Y537S	0.376441	3	
MET_22	PIK3CA	E453K	0.444722	3	Buparlisib, serabelisib, alpelisib + fulvestrant, copanlisib, GDC-0077, alpelisib, taselisib + fulvestrant, buparlisib + fulvestrant, taselisib
MET_10	PIK3CA	E542K	0.106212	3	
MET_21	PIK3CA	E542K	0.501912	3	
MET_41	PIK3CA	E542K	0.073183	3	
MET_49	PIK3CA	E542K	0.467702	3	
MET_08	PIK3CA	E545K	0.204327	3	
MET_34	PIK3CA	E545K	0.0871914	3	
MET_40	PIK3CA	E545K	0.844344	3	
MET_25	PIK3CA	H1047R	0.341171	3	
MET_29	PIK3CA	H1047R	0.180681	3	
MET_32	PIK3CA	H1047R	0.2785	3	
MET_33	PIK3CA	H1047R	0.413998	3	
MET_38	PIK3CA	H1047R	0.384692	3	
MET_44	PIK3CA	H1047R	0.60054	3	
MET_06	PIK3CA	N345K	0.376571	3	
MET_35	PIK3CA	Q546R	0.435484	3	
PR_26	BRAF	G469A	0.52028	4	LTT462, BVD-523, KO-994
MET_34	KRAS	G12D	0.074	4	LY3214996, KO-947, GDC-1014
MET_22	PTEN	C136Y	0.756233	4	AZD6482 + alpelisib
CF_28_Draw_1	ESR1	D538G	0.0746562	3	AZD9496, fulvestrant
CF_28_Draw_5	ESR1	D538G	0.146853	3	
CF_22_Draw_1	PIK3CA	H1047R	0.320088	3	Buparlisib, serabelisib, alpelisib + fulvestrant, copanlisib, GDC-0077, alpelisib, taselisib + fulvestrant, buparlisib + fulvestrant, taselisib
CF_22_Draw_2	PIK3CA	H1047R	0.402402	3

### Characterization of genetic variants detected by MammaSeq in ctDNA

To examine the potential of MammaSeq™ to detect variants in ctDNA, we sequenced 14 ctDNA samples isolated from 7 patients with metastatic disease, this cohort being independent of the solid tumor cohort above. ctDNA samples were sequenced to a mean depth of 1810×, while patient-matched buffy coat gDNA was sequenced to a mean depth of 425× (Additional file 6: Figure S2).

**Fig. 4:**
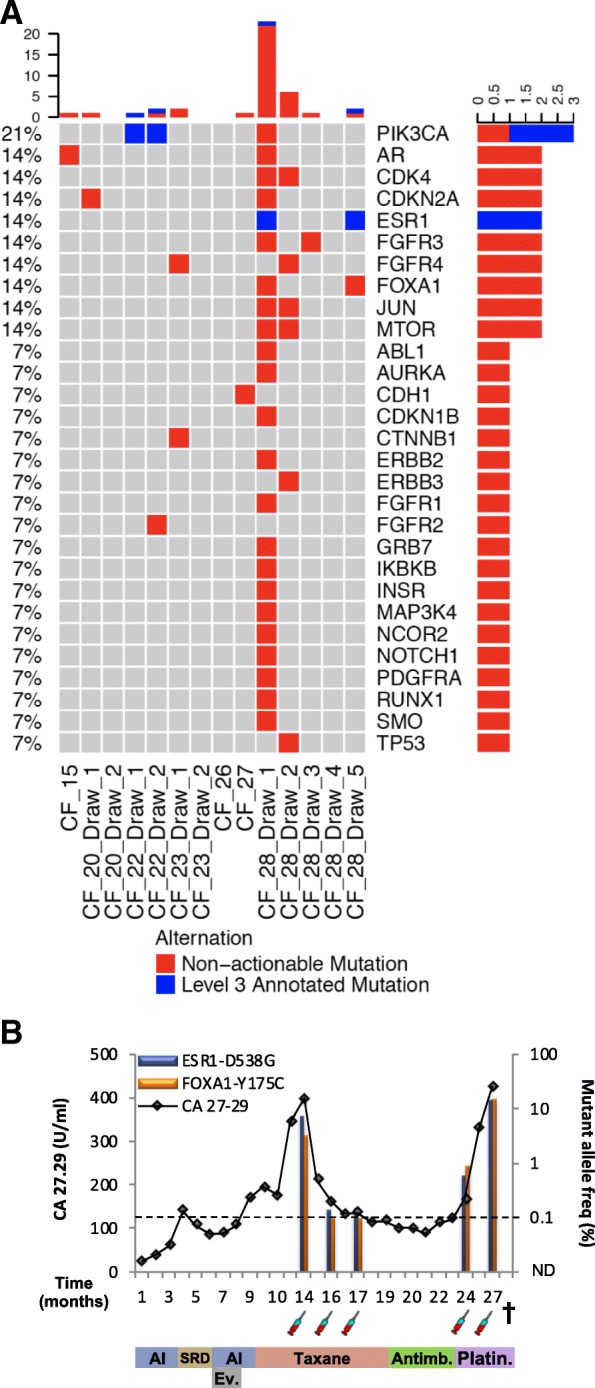
Genetic alterations identified in ctDNA from a test cohort of 7 patients with metastatic invasive ductal carcinoma. **a** Oncoprint of somatic mutations identified in 14 ctDNA samples. **b** Clinical timeline and mutant allele frequency of ESR1-D538G and FOXA1-Y175C mutations in serial blood draws from patient CF28. The timeline starts with diagnosis of metastasis and shows tumor marker assessments (CA 27.29 antigen line graph), mutant allele frequency (bar graphs), LLoD (dotted line), blood draws (syringe), and treatments received. Treatment abbreviations: AI (aromatase inhibitor), SERD (selective estrogen receptor degrader), Ev. (everolimus), Antimb. (antimetabolite), Platin (platinum-based chemotherapy)

Variants were called on ctDNA and gDNA, and patient-matched variants present in both (i.e., germline variants) were removed. We then applied the same filtering pipeline to the ctDNA variants and solid tumor variants; except in this smaller cohort, we removed all identical variants found in more than 4 samples (as these are likely platform specific sequencing errors) and lowered the minimum allele frequency to 1.0% to increase detection rate in this research cohort. We identified a total of 43 somatic mutations across the 14 ctDNA samples (mean 3.1, median 1, IQR 1.75) (Fig. 4a, Additional file 10: Data file 3). Similar to the solid tumor cohort, a single draw from 1 patient (CF_28-Draw 1) harbored 13 of the 25 (58%) total mutations. Using the same definition, this sample is also an outlier. Similar to the solid tumor cohort, PIK3CA and ESR1 were among the most commonly mutated genes.

Two of the identified somatic mutations (each identified in 2 draws from 1 patient) are annotated at level 3 in the OncoKB database, ESR1-and PIK3CA-H1047R (Fig. 4a). The ESR1 mutation was identified in 2 separate blood draws from patient CF_28 taken 13 months apart. Interestingly, the FOXA1-Y175C mutation was also identified in the same draws from patient CF_28 (Fig. 4b). The allele frequencies of the ESR1 and FOXA1 mutations, but not the total number of mutations, strongly correlated with levels of cancer antigen 27-29 (CA-27.29). Mutations identified in all three genes (ESR1, PIK3CA, and FOXA1) were independently validated using ddPCR (Additional file 11: Figure S5).

## Discussion

Advances in the accuracy, cost, and analysis of NGS make it an ideal platform to develop diagnostics that can be used to precisely identify treatment options. MammaSeq was developed to comprehensively cover known driver mutation hotspots specifically in primary and metastatic breast cancer that would identify mutations with potential prognostic value. Typically, NGS diagnostics are reserved for late-stage disease. As a result, the solid tumor cohort was significantly enriched for metastatic disease and markers of poor prognosis—triple negative subtype, late presentation, and therapy resistance [8], whereas the ctDNA cohort was mainly ER+ disease. As such, different mutations were found in each cohort, e.g., TP53 mutations were common in the solid tumor cohort but rare in the ctDNA likely due to the differences in breast cancer subtype.

Consistent with previous mutational studies, we report that a small subset of breast cancers harbor high mutational burden [26]. Across a variety of cancers, groups have demonstrated the correlation between the tumor mutational burden (TMB) and the efficacy of immunotherapy checkpoint inhibitors (reviewed here [27]). However, the ability to accurately depict tumor mutational burden is dependent on the percentage of the covered exome. A study by Chalmers et al. used a computational model to show that below 0.5 Mb, TMB measurements are highly variable and unreliable [28]. The MammaSeq™ panel covers just 82,035 bp (0.08 Mb) and therefore likely cannot be used to calculate a mutational burden comparable to whole exome-based studies. That being said, the stark difference in the total number of mutations identified in the subset of 4 tumor samples suggests a high TMB, meaning these patients may be suited for immunotherapy.

Limitations of this study include the small cohort of patients, the observational nature of the cohort that limits association of mutations with outcome, the inability to completely capture all mutations given rapid advances in the field, and the potential for false-positive results given challenges with detection of rare events, particularly in ctDNA. We also note that there were different breast cancer subtypes in the solid and ctDNA cohorts. Despite these limitations, we believe the pilot study shows that the MammaSeq panel is useful for researchers to rapidly and cost-effectively detect somatic mutations in solid tumors and ctDNA.

Liquid biopsies are beginning to be utilized clinically after numerous proof-of-principle studies have demonstrated the potential of circulating tumor DNA (ctDNA) for prognostication, molecular profiling, and monitoring disease burden [13, 29–33]. We have demonstrated that the MammaSeq™ panel can be used to identify mutations in ctDNA. For one patient (CF_28), we have ctDNA data from 5 blood draws taken over the course of 13 months. The sharp drop-off in the number of somatic mutations identified between the first and second draws co-occurs with a decrease in CA.27.29 levels, suggesting that the patient may have responded well to treatment, leading to disappearance of sensitive clones. In the later blood draws, we did not observe an increase in the total number of somatic mutations, but instead an increase in the allele frequency of ESR1-D538G and FOXA1-Y175C mutations, which may suggest therapeutic selection of resistant clones. Numerous studies are currently examining longitudinal changes in mutations in ctDNA, and a recent comprehensive analysis using whole exome sequencing in non-small cell lung cancer revealed diverse changes in mutations over time [34].

High-throughput genotyping of solid tumors and continual monitoring of disease burden through sequencing of ctDNA represent potential clinical applications for NGS technologies. The detection of rare events is challenging due to the false detection rate of different NGS platforms. We have used extra deep sequencing to reduce the false-positive rate, but alternative approaches such as inclusion of UMIs can be utilized. More research is required to enhance the sensitivity and specificity of mutation detection in ctDNA. We note that the 1% allele fraction cutoff we used in the ctDNA analysis is for research purposes to increase sensitivity, and this is not clinically validated. It should be noted that targeted DNA sequencing panels such as MammaSeq™ are far less comprehensive than whole exome sequencing and they do not allow for evaluation of structural variants, which may lead to gene fusions that function as drivers [35]. However, the small panel combined with amplification-based sequencing allows for detection in very small amounts of DNA (10 ng) and thus is suitable for small biopsies. Focused panels represent cost-effective and useful alternatives to whole exome sequencing for targeted mutation detection.

## Conclusions

Here we report the development of MammaSeq™, a targeted sequencing panel designed based on current knowledge of the most common, impactful, and targetable drivers of metastatic breast cancer. This data provides further evidence for the use of NGS diagnostics in the management of advanced breast cancers.

## Additional files

Additional file 1: **Table S1.** Detailed patient clinical information. (PDF 252 kb)

Additional file 2: Data file 1 Genomic location of mutations in MammaSeq panel. (XLSX 113 kb)

Additional file 3: **Table S2.** Custom-designed primers for preamplification. (PDF 122 kb)

Additional file 4: **Table S3.** Custom-designed ddPCR primers (PDF 148 kb)

Additional file 5: **Figure S1.** MammaSeq™ gene coverage. The percentage of protein coding bases pairs in each gene that is sequenced by the MammaSeq™ panel. (PDF 79 kb)

Additional file 6: **Figure S2.** Mean sequencing read depth for (A.) the 46 solid tumor cohort. (B.) isolated mononuclear cells from the 14 ctDNA draws and (C.) the 14 ctDNA samples. (PDF 191 kb)

Additional file 7: Data file 2 Single nucleotide variants detected by MammaSeq in solid tumors. (XLSX 255 kb)

Additional file 8: **Figure S3.** Correlation between variant allele frequencies detected by Cancer Hotspot Panel V2 and MammaSeq. (PDF 96 kb)

Additional file 9: **Figure S4.** Tumor mutational burden across all samples in the 46 solid tumor cohort. (A.) Total detected mutations for each sample. (PDF 40 kb)

Additional file 10: Data file 3 Single nucleotide variants detected by MammaSeq in cfDNA. (XLSX 60 kb)

Additional file 11: **Figure S5.** ddPCR validation of mutations identified by MammaSeq™ is indicated along with mutant allele frequencies for (A.) ESR1-D538G, (B.) FOXA1-Y175C, and (C.) PIK3CA-H1047R. (PDF 1562 kb)

## Abbreviations

AF: Allele frequency
CNV: Copy number variants
ctDNA: Circulating-tumor DNA
ddPCR: Droplet digital PCR
dgIDB: Drug-Gene Interaction Database
gDNA: Germline DNA
GO: Gene Ontology
GWAS: Genome-wide association studies
IDC: Invasive ductal carcinoma
ILC: Invasive lobular carcinoma
ISP: Ion sphere particles
MBC: Metastatic breast cancer
NGS: Next-generation sequencing
PGM: Ion Torrent Personal Genome Machine
SMG: Significantly mutated gene
SNP: Single nucleotide polymorphism
SNV: Single nucleotide variant
TCGA: The Cancer Genome Atlas
TMB: Tumor mutational burden
