# Supplementary material for: Targeted mutation detection in breast cancer using MammaSeq™
Source: Breast Cancer Res. 2019 Feb 8;21:22. doi: 10.1186/s13058-019-1102-7 (PMC6368740; doi:10.1186/s13058-019-1102-7)
Supplement: Supplementary file 1 — Table S1. Detailed patient clinical information. (PDF 252 kb) [file 13058_2019_1102_MOESM1_ESM.pdf]

Table S1. Clinical characteristics, treatment, and outcome data for all patients.

Patient ID for manuscript	Age at Diagnosis	Race 1-Desc	Histo/Behavior ICD-O-3-Desc	Grade/Differentiation	Size of Tumor	Clinical T	Clinical N	Clinical M	Clinical Stage Group	Pathologic T	Pathologic N	Pathologic M	Pathologic Stage Group	ER	PR	HER2	Vital Status-Desc	Disease-Free Survival	Months from Dx to 1st Recur	Survival
PR02	43	White	IDC	3	N/A	N/A	N/A	N/A	N/A	N/A	N/A	N/A	N/A	-	-	-	N/A	N/A	N/A	N/A
PR11	42	Black	IDC/ILC	3	72	c3	c0	c0	2B	p1C	p0	N/A	1A	-	-	-	Dead	15	17	22
PR14	41	White	IDC	3	17	c1C	c0	c0	1A	p1C	p1	N/A	2A	+	+	-	Dead	19	19	46
PR19	49	White	IDC	N/A	N/A	N/A	N/A	N/A	N/A	N/A	N/A	N/A	N/A	-	-	-	Dead	0	0	0
PR24	51	White	IDC	3	70	c2	c1	c0	2B	p4	p3A	N/A	3C	+	+	+	Dead	0	0	47
PR26	54	White	IDC	N/A	N/A	N/A	N/A	N/A	N/A	N/A	N/A	N/A	N/A	+	+	-	Dead	0	0	0
PR31	54	White	IDC	3	42	c2	c1	c0	2A	p1B	p1A	N/A	2A	+	+	-	Dead	15	15	37
PR39	64	White	IDC	2	60	c1C	c1	c0	2A	p3	p2A	N/A	3A	+	+	+	Alive	3	5	54
PR43	65	White	IDC	2	31	c2	c0	c0	2B	p2	p2A	N/A	3A	+	+	-	Dead	12	14	36
PR48	70	White	IDC	3	N/A	N/A	N/A	N/A	N/A	p4A	p2A	c0	3B	+	+	-	Dead	7	7	47
MET01	30	White	IDC	3	046	c2	c1	c0	2B	p2	p3A	N/A	3C	-	-	-	Dead	16	16	26
MET03	39	White	N/A	4	N/A	c4D	c0	c0	3B	pIS	p0	c0	0	-	-	+	Alive	92	92	127
MET05	32	White	IDC	3	065	c3	c3	c0	3C	p3	p3	N/A	3C	-	-	-	Dead	8	8	40
MET06	31	White	IDC	3	38	c2	c1A	c0	2B	p1B	p1A	c0	1	+	-	-	Dead	13	13	66
MET07	36	White	IDC	3	33	c2	c0	c1	4	p1C	p1A	p1	4	+	-	-	Dead	0	0	55
MET08	36	White	IDC	2	31	c2	c0		2A	p2	p2A		3A	+	+	-	Dead	32	33	61
MET09	38	White	IDC	3	80	c3	c1	c0	3A	pIS	p0		0	-	-	-	Dead	15	15	20
MET10	37	White	IDC	9	10	c1	c1	c0	2A	p1	p1		2A	+	-	-	Dead	52	52	67
MET12	42	White	IDC	3	20	c1C	c1	c0	2B	p1B	p0		1A	-	-	-	Dead	12	13	42
MET13	42	White	IDC	3	61	c3	c1	c0	3A	p2	p2A		3A	-	-	-	Dead	20	21	29
MET15	-	White	N/A	9	999								N/A	-	-	-	Dead	0	0	0
MET16	37	White	ILC	2	100	c3	c1	c0	3A	p1	p2A		3A	+	-	-	Dead	82	84	138
MET17	-	White	IDC	9	999								N/A	N/A	N/A	N/A	Alive	0	0	0
MET18	45	White	IDC	3	120	c3	c2	c1	4	p3	p1A	p1	4	-	-	-	Dead	0	0	25
MET20	47	White	ILC	3	58	c3	c1	c1	4	pX	pX		N/A	+	+	-	Dead	0	0	25
MET21	35	White	IDC	3	13	c1	c0	c0	1	p1C	p0	c0	1	+	+	+	Alive	32	33	221
MET22	36	White	N/A	9	13	cX	cX	c0	99	p1C	p0		1	-	-	-	Dead	84	84	181
MET23	47	White	IDC/ILC	3	35	c2	c1	c0	2B	p1B	p0		1A	-	-	-	Dead	25	25	63
MET25	42	White	IDC/ILC	2	50	c1	c0	c0	1	p2	p1	c0	2B	+	+	-	Dead	123	125	163
MET27	49	White	IDC/ILC	3	90	c3	c1	c0	3A	p2	p3	c0	3C	+	+	-	Dead	28	28	68
MET28	38	White	IDC	9	14	c1	c0	c0	1	p1C	pX	c0	N/A	+	+	+	Dead	63	64	213
MET29	54	White	IDC	3	999	c4B	c1		3B	pX	pX		N/A	-	-	-	Dead	0	0	18
MET30	45	White	IDC	9	60	c3	c1	c0	3A	p1C	p1	c0	2A	+	+	-	Dead	87	88	146
MET32	54	White	IDC	3	34	c2	c1	c1	4	p2	p0	p1	4	+	+	-	Alive	0	0	68
MET33	31	White	IDC	9	999	c1	c0	c0	1	p1	p0		1	+	-	+	Dead	274	274	348
MET34	45	White	IDC	2	030	c2	c1	c0	2B	p2	p0	c0	2A	N/A	N/A	N/A	Dead	42	43	201
MET35	48	White	ILC	9	120	c4B	c1	c0	3B	p3	p1	c0	3A	+	+	-	Dead	135	136	170
MET36	58	White	IDC	3	23	c2	c0	c0	2A	pX	pX		N/A	-	-	-	Dead	15	16	41
MET37	60	White	IDC	9	30	c2	cX	c1	4	p2	p3A	p1	4	-	+	-	Dead	0	0	26
MET38	45	White	ILC	9	42	c2	c0	c0	2A	p2	p1B	c0	2B	+	-	-	Dead	198	198	226
MET40	57	White	IDC	2	28	cX	cX	cX	99	p2	p1A	c0	2B	-	-	-	Dead	86	86	93
MET41	67	White	ILC	9	2	c1A	c0	c1	4	pX	pX		N/A	+	N/A	N/A	Alive	0	0	34
MET44	-	White	N/A	9	999								N/A	-	-	-	Dead	0	0	0
MET46	68	White	IDC	2	18	c1C	c0	c0	1A	p1C	p0		1A	+	-	-	Alive	4	6	40
MET47	69	White	IDC	3	12	cX	c0	c0	99	p1C	p0		1A	-	-	-	Alive	19	19	22
MET49	71	White	IDC	3	19	c2	c0	c0	2A	p1C	p0		1A	+	+	-	Dead	44	44	51
CF15	24	white	IDC	2	997	3	1	0	3A	1A	1	0	2A	+	+	N/A	1	79	79	138
CF20	53	white	IDC	3	074	3	1	0	3A	2	1A	0	2B	+	+	N/A	1	64	64	96
CF22	34	white	IDC	2	025	N/A	N/A	N/A	99	N/A	N/A	N/A	99	+	+	N/A	1	0	0	0
CF23	54	white	IDC	3	034	2	1	1	4	2	0	1	4	+	N/A	N/A	1	0	0	40
CF26	62	white	IDC	9	013	1C	3	0	3B	X	X	X	99	+	N/A	N/A	1	151	152	224
CF27	42	white	IDC	3	074	3	3	1	4	1C	3	1	4	+	+	N/A	0	0	0	80
CF28	55	white	IDC	2	018	1C	0	0	1	1C	1M	0	2A	+	+	N/A	1	116	117	124
