# Supplementary material for: Targeted mutation detection in breast cancer using MammaSeq™
Source: Breast Cancer Res. 2019 Feb 8;21:22. doi: 10.1186/s13058-019-1102-7 (PMC6368740; doi:10.1186/s13058-019-1102-7)
Supplement: Supplementary file 3 — Table S2. Custom-designed primers for preamplification. (PDF 122 kb) [file 13058_2019_1102_MOESM3_ESM.pdf]

**Table S2. Sequence of primers for preamplification.**

| Mutation | Forward primer | Reverse primer |
|---|---|---|
| ESR1-D538G | GCATGAAGTGCAAGAACGTG | AAGTGGCTTTGGTCCGTCT |
| FOXA1-Y175C | TGGATGGCCATGGTGATGAG | AGACGTTCAAGCGCAGCTA |
