# Supplementary material for: Targeted mutation detection in breast cancer using MammaSeq™
Source: Breast Cancer Res. 2019 Feb 8;21:22. doi: 10.1186/s13058-019-1102-7 (PMC6368740; doi:10.1186/s13058-019-1102-7)
Supplement: Supplementary file 4 — Table S3. Custom-designed ddPCR primers (PDF 148 kb) [file 13058_2019_1102_MOESM4_ESM.pdf]

Table S3. Sequence of ddPCR primers and probes.

Mutation	Forward primer	Reverse primer	Mutant Probe	WT probe	Fluorescence
ESR1-D538G	GCATGAAGTGCAAGAACGTG	AAGTGGCTTTGGTCCGTCT	TCTATGGCCTGCTGCTGGAGATGCT	TCTATGACCTGCTGCTGGAGATGCT	HEX/FAM
FOXA1-Y175C	TGGATGGCCATGGTGATGAG	AGACGTTCAAGCGCAGCTA	CTACTCGTGCATCTCG	CCTACTCGTACATCTCG	FAM/VIC
