# Supplementary figures and images for: Targeted mutation detection in breast cancer using MammaSeq™
Source: Breast Cancer Res. 2019 Feb 8;21:22. doi: 10.1186/s13058-019-1102-7 (PMC6368740; doi:10.1186/s13058-019-1102-7)

### Figure S1

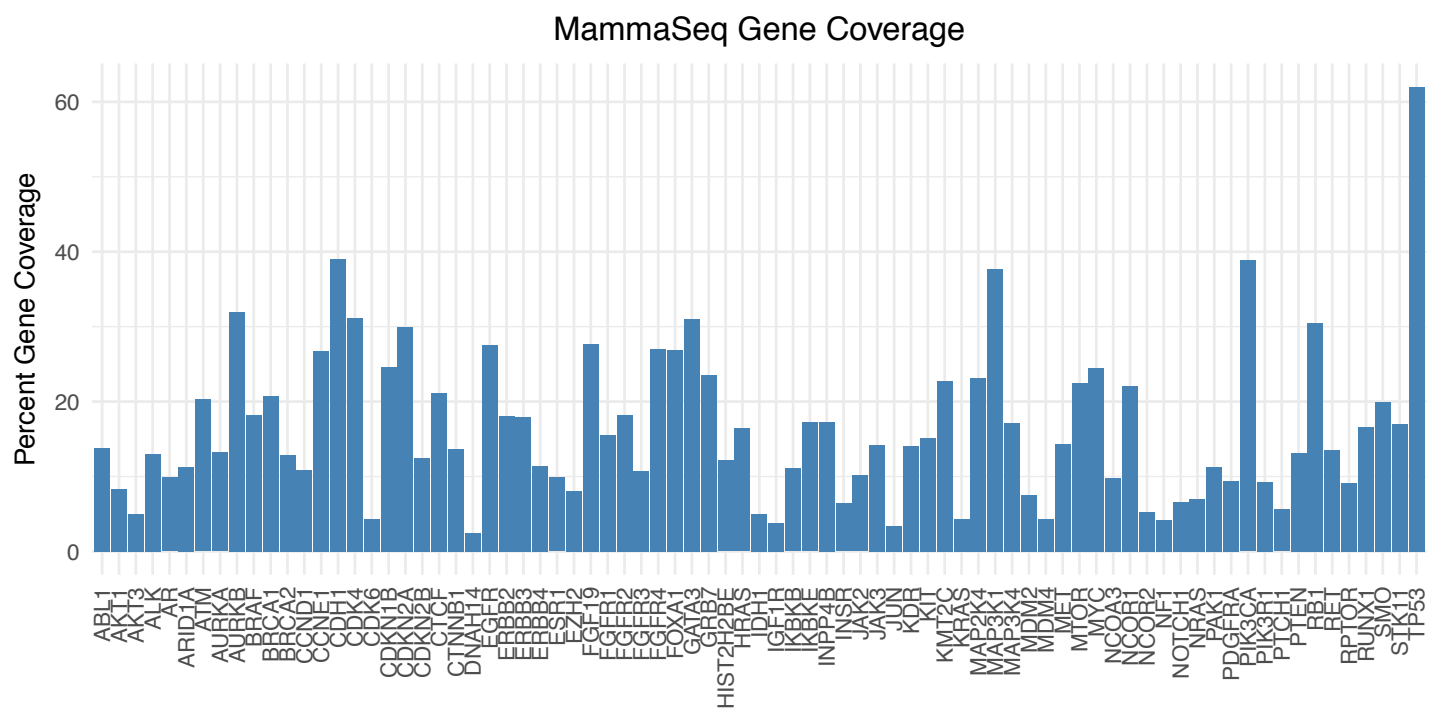

Supplement: Supplementary file 5 — Figure S1. MammaSeq™ gene coverage. The percentage of protein coding bases pairs in each gene that is sequenced by the MammaSeq™ panel. (PDF 79 kb) [file 13058_2019_1102_MOESM5_ESM.pdf]

Figure S2

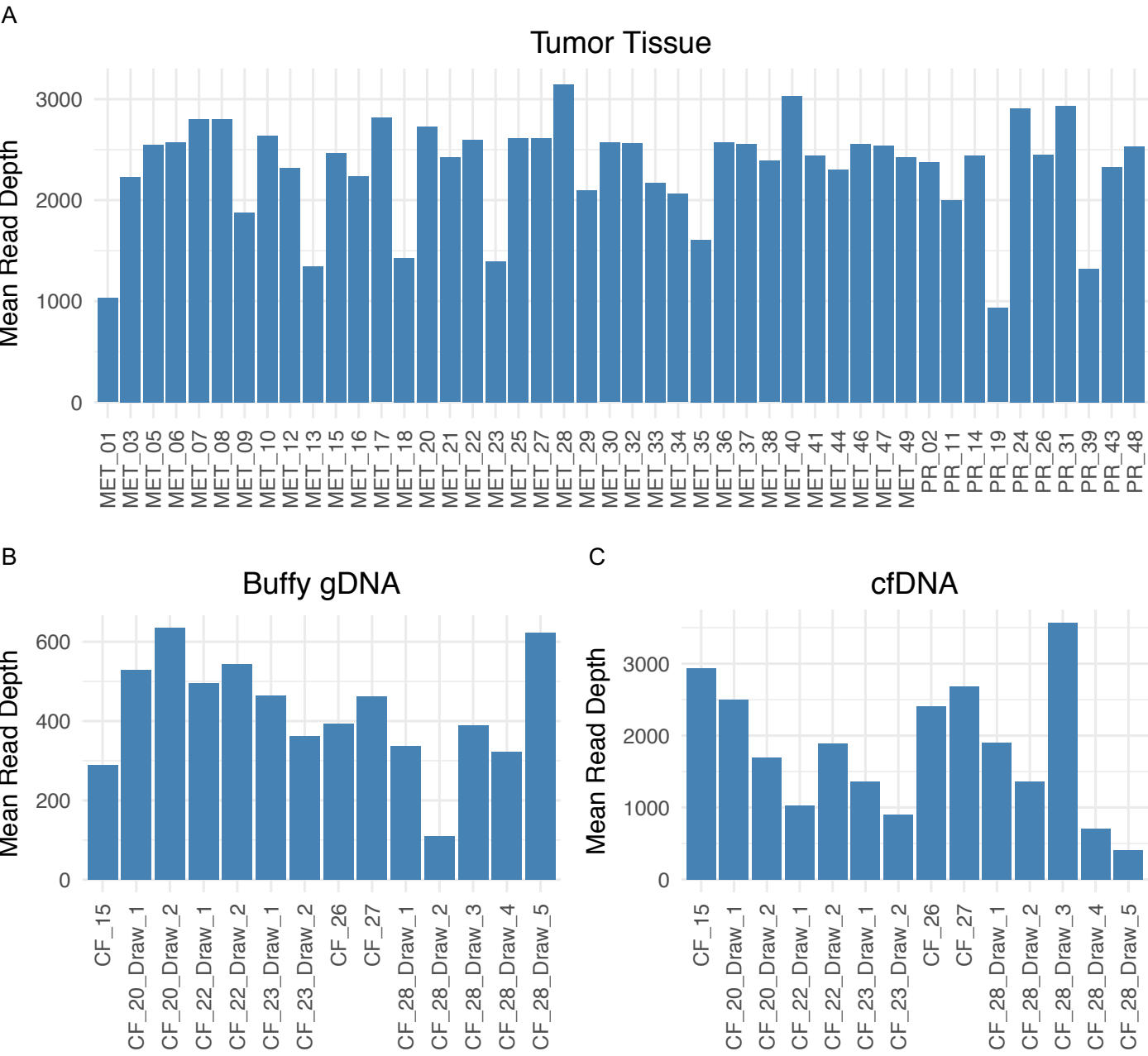

Supplement: Supplementary file 6 — Figure S2. Mean sequencing read depth for (A.) the 46 solid tumor cohort. (B.) isolated mononuclear cells from the 14 ctDNA draws and (C.) the 14 ctDNA samples. (PDF 191 kb) [file 13058_2019_1102_MOESM6_ESM.pdf]

Figure S3

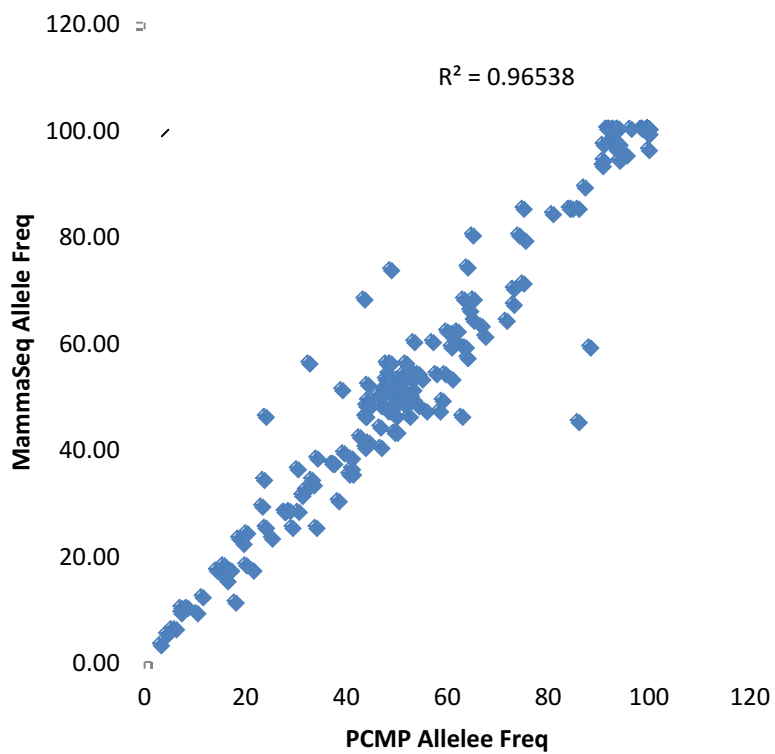

Supplement: Supplementary file 8 — Figure S3. Correlation between variant allele frequencies detected by Cancer Hotspot Panel V2 and MammaSeq. (PDF 96 kb) [file 13058_2019_1102_MOESM8_ESM.pdf]

Supplemental Figure 4

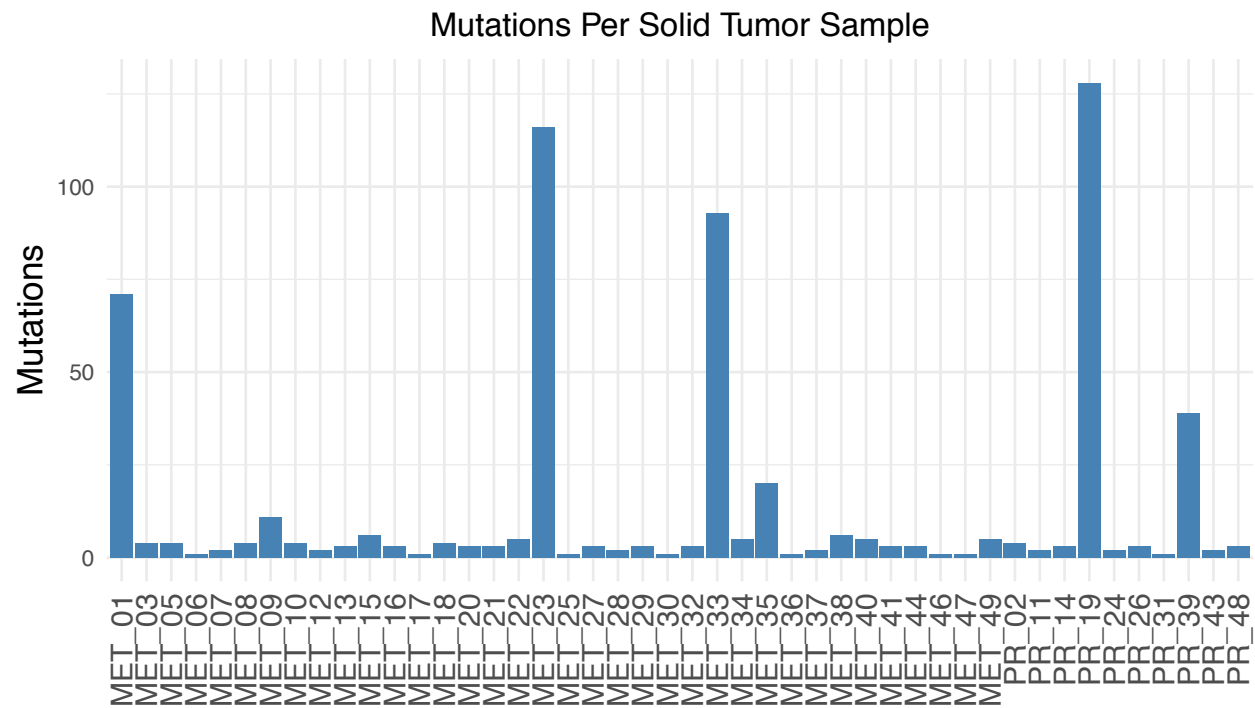

Supplement: Supplementary file 9 — Figure S4. Tumor mutational burden across all samples in the 46 solid tumor cohort. (A.) Total detected mutations for each sample. (PDF 40 kb) [file 13058_2019_1102_MOESM9_ESM.pdf]

Figure S5

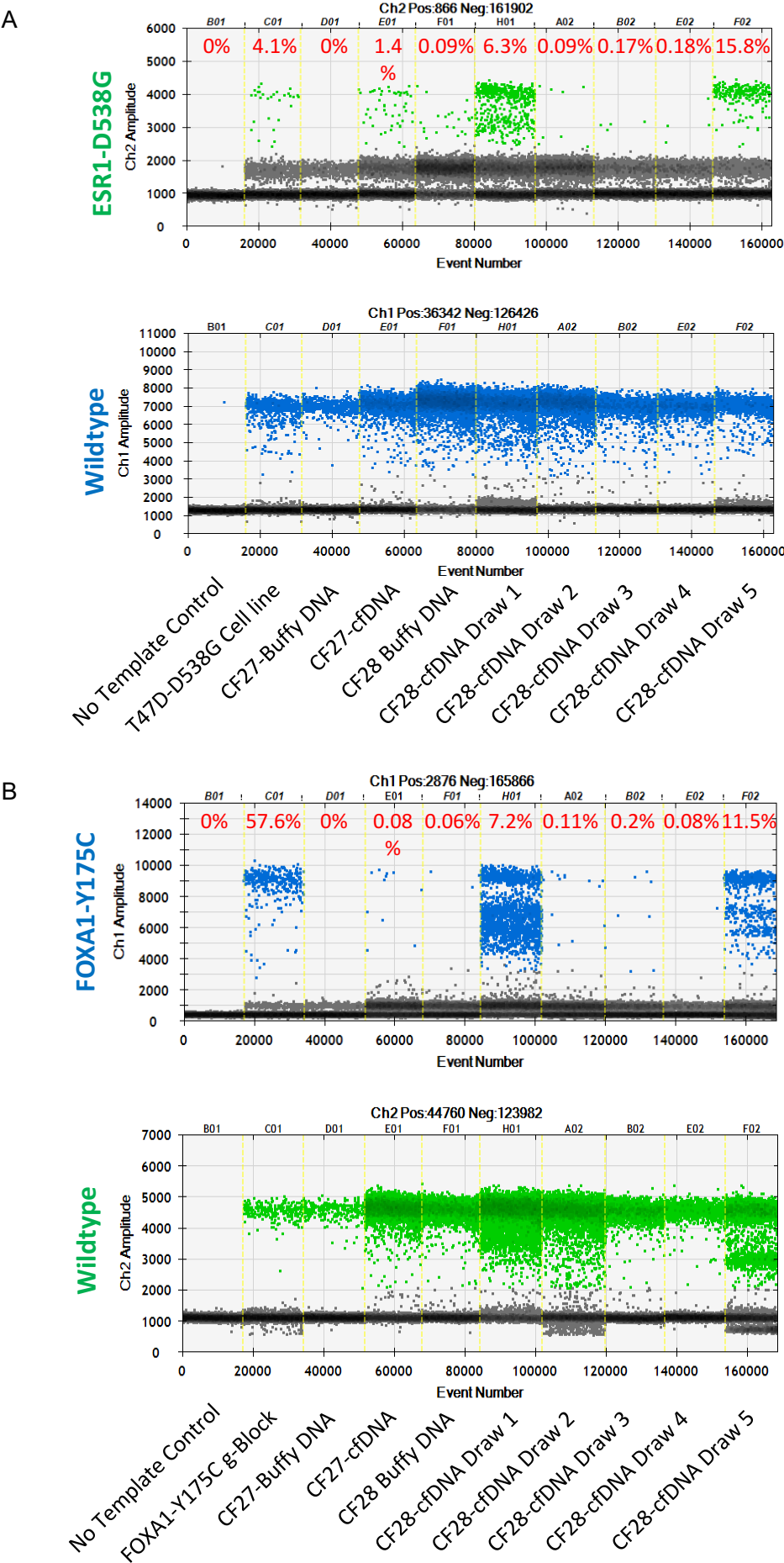

C

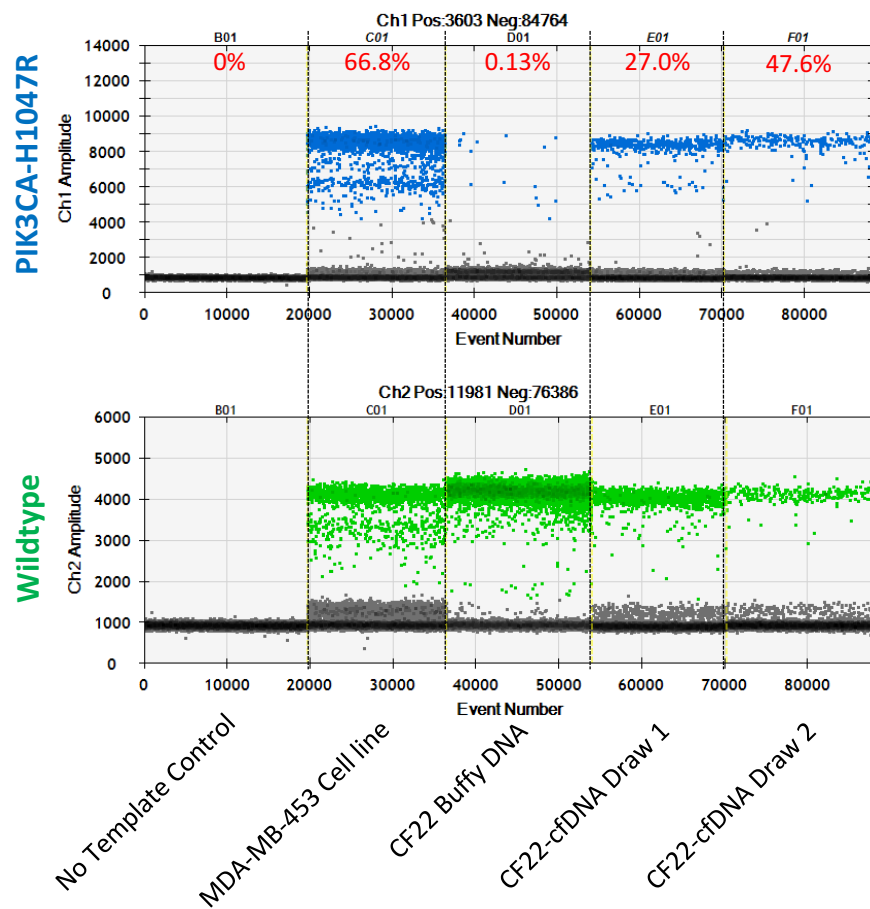

Supplement: Supplementary file 11 — Figure S5. ddPCR validation of mutations identified by MammaSeq™ is indicated along with mutant allele frequencies for (A.) ESR1-D538G, (B.) FOXA1-Y175C, and (C.) PIK3CA-H1047R. (PDF 1562 kb) [file 13058_2019_1102_MOESM11_ESM.pdf]
